# Association between class of foundational medication for heart failure and prognosis in heart failure with reduced/mildly reduced ejection fraction

**DOI:** 10.1038/s41598-022-20892-3

**Published:** 2022-10-05

**Authors:** Miyuki Ito, Daichi Maeda, Yuya Matsue, Yasuyuki Shiraishi, Taishi Dotare, Tsutomu Sunayama, Kazutaka Nogi, Makoto Takei, Tomoya Ueda, Maki Nogi, Satomi Ishihara, Yasuki Nakada, Rika Kawakami, Nobuyuki Kagiyama, Takeshi Kitai, Shogo Oishi, Eiichi Akiyama, Satoshi Suzuki, Masayoshi Yamamoto, Keisuke Kida, Takahiro Okumura, Yuji Nagatomo, Takashi Kohno, Shintaro Nakano, Shun Kohsaka, Tsutomu Yoshikawa, Yoshihiko Saito, Tohru Minamino

**Affiliations:** 1 Department of Cardiovascular Biology and Medicine, Juntendo University Graduate School of Medicine, 3-1-3 Hongo, Bunkyo-ku, Tokyo Japan; 2 Division of Cardiology, Department of Medicine, Keio University School of Medicine, Tokyo, Japan; 3 Department of Cardiovascular Medicine, Nara Medical University, Kashihara, Japan; 4 Department of Cardiology, Tokyo Saiseikai Central Hospital, Tokyo, Japan; 5 Department of Cardiology, Saiseikai Suita Hospital, Suita, Japan; 6 Department of Cardiovascular Medicine, National Cerebral and Cardiovascular Center, Osaka, Japan; 7 Department of Cardiology, Himeji Cardiovascular Center, Himeji, Japan; 8 Division of Cardiology, Yokohama City University Medical Center, Yokohama, Japan; 9 Department of Cardiology and Hematology, Fukushima Medical University, Fukushima, Japan; 10 Cardiovascular Division, Faculty of Medicine, University of Tsukuba, Tsukuba, Japan; 11 Department of Pharmacology, St. Marianna University School of Medicine, Kanagawa, Japan; 12 Department of Cardiology, Nagoya University Graduate School of Medicine, Nagoya, Japan; 13 Department of Cardiology, National Defense Medical College, Tokorozawa, Japan; 14 Department of Cardiovascular Medicine, Kyorin University School of Medicine, Tokyo, Japan; 15 Department of Cardiology, International Medical Center, Saitama Medical University, Saitama, Japan; 16 Department of Cardiology, Sakakibara Heart Institute, Tokyo, Japan; 17 Japan Agency for Medical Research and Development-Core Research for Evolutionary Medical Science and Technology (AMED-CREST), Japan Agency for Medical Research and Development, Tokyo, Japan; 18 Cardiovascular Respiratory Sleep Medicine, Juntendo University Graduate School of Medicine, 3-1-3 Hongo, Bunkyo-ku, Tokyo Japan; 19 Nara Prefectural Hospital Organization, Nara Prefecture Seiwa Medical Center, Nara, Japan

**Keywords:** Heart failure, Combination drug therapy

## Abstract

We clarified the association between changes in the number of foundational medications for heart failure (FMHF) during hospitalization for worsening heart failure (HF) and post-discharge prognosis. We retrospectively analyzed a combined dataset from three large-scale registries of hospitalized patients with HF in Japan (NARA-HF, WET-HF, and REALITY-AHF) and patients diagnosed with HF with reduced or mildly reduced left ventricular ejection fraction (HFr/mrEF) before admission. Patients were stratified by changes in the number of prescribed FMHF classes from admission to discharge: angiotensin-converting enzyme inhibitors or angiotensin receptor blockers, beta-blockers, and mineralocorticoid receptor blockers. Primary endpoint was the combined endpoint of HF rehospitalization and all-cause death within 1 year of discharge. The cohort comprised 1113 patients, and 482 combined endpoints were observed. Overall, FMHF prescriptions increased in 413 (37.1%) patients (increased group), remained unchanged in 607 (54.5%) (unchanged group), and decreased in 93 (8.4%) (decreased group) at discharge compared with that during admission. In the multivariable analysis, the increased group had a significantly lower incidence of the primary endpoint than the unchanged group (hazard ratio 0.56, 95% confidence interval 0.45–0.60; *P* < 0.001). In conclusion, increase in FMHF classes during HF hospitalization is associated with a better prognosis in patients with HFr/mrEF.

## Introduction

Heart failure (HF) is a major public health burden worldwide that is associated with high medical costs and unsatisfactory prognoses. Although multiple medications have improved the prognosis of patients with HF with reduced left ventricular ejection fraction (HFrEF)^1–3^, there remains substantial gaps in clinical practice in terms of implementation^4,5^.

Among patients with HF who require hospitalization because of worsening symptoms and high risk of future adverse events^6,7^, hospital admission offers an important opportunity to optimize HF treatment^8^. Current guidelines recommend introducing each class of foundational medications for HF (FMHF) to patients, subsequently titrating the dose to the target dose, and then introducing another class of FMHF if the patient does not respond well to the treatment. Although this sequential optimization of FMHF has been well accepted in daily clinical practice, several studies have indicated that adding another class of FMHF even at low doses may outweigh uptitrating the dose of FMHF already prescribed^9–12^. In this sense, an increase in the number of FMHF classes during hospitalization for worsening HF may be associated with better prognosis. However, although previous studies have demonstrated that a higher number of FMHF classes prescribed at the time of discharge is associated with better post-discharge prognosis^13,14^, no study has evaluated the association between changes in the number of FMHF classes and prognosis in patients requiring hospitalization for worsening HF.

Therefore, we aimed to clarify the modification pattern of FMHF during hospitalization due to worsening HF in patients already diagnosed with HF and its impact on outcomes using real-world multicenter prospective registry data.

## Methods

### Study design

This study was conducted using three Japanese prospective hospital-based registries for acute HF, i.e., NARA-HF, WET-HF, and REALITY-AHF. All study participants were admitted to each participating hospital. The detailed study design of each registry has been reported previously^15–17^. All three registries included patients hospitalized with acute HF, regardless of the left ventricular ejection fraction (LVEF). Patients with acute coronary syndrome were excluded from all three registries. The study protocols of all three registries were approved by the institutional review boards of each site, and all studies were conducted in compliance with the tenets of the Declaration of Helsinki^15,17,18^. The present study that aimed to analyze the dataset combining the three registries was approved by the Nara Medical University Institutional Ethics Committee (No. 2456) and was performed in accordance with the 1975 Declaration of Helsinki guidelines for clinical research protocols. Informed consent was obtained from all patients.

### Study registries

We combined and analyzed three registries (REALITY-AHF, NARA-HF, and WET-HF) in this study. Further information on these registries is available in Supplementary Table S1.

### Collection of clinical data and definitions used in the current study

The diagnosis of acute HF was based on the Framingham criteria in all registries^19^. In REALITY-AHF, patients with a brain natriuretic peptide (BNP) level < 100 pg/mL or N-terminal-proBNP level < 300 pg/mL at baseline were excluded^2,20–22^. Baseline data, including age, sex, and medical history, were obtained on admission. Blood samples and echocardiographic findings were obtained in a clinically compensated state prior to discharge. Information on oral medications, including angiotensin-converting enzyme inhibitor (ACEi)/angiotensin II receptor blocker (ARB), beta-blocker (BB), and mineralocorticoid receptor antagonist (MRA), was obtained at both admission and discharge.

In the current analysis, we excluded patients with missing data on ACEi/ARB, BB, or MRA at admission, discharge, or both, those with an LVEF ≥ 50%; and those without a history of HF. A history of HF was defined as a previous hospitalization for HF before index hospitalization.

### Groups

Patients were divided into three groups according to the modification pattern of FMHF (ACEi/ARB, BB, and MRA) during index hospitalization. When the number of FMHF classes increased, decreased, or remained unchanged from admission to discharge, patients were classified into the increased, decreased, or unchanged groups, respectively. For instance, patients for whom medications were modified from ACEi to ACEi + BB were classified under the “increased” group. Similarly, patients for whom medications were modified from ARB + MRA to ACEi + BB were classified under the “unchanged” group.

### Clinical outcomes

The primary endpoint of the current study was a composite of all-cause death or rehospitalization due to HF at 1 year after discharge.

### Statistical analysis

Continuous variables are described as means ± standard deviations or as medians with interquartile ranges and were compared using Student's t-test or Mann–Whitney U test, as appropriate. Categorical variables are reported as numbers with percentages and were compared using the χ^2^ or Fisher exact test, as appropriate.

Cumulative event rates between the groups were compared using the Kaplan–Meier method with the log-rank test. Unadjusted and adjusted Cox proportional hazard analyses were performed to evaluate the association between the modification pattern and prognosis. The following variables were included in the adjusted model since these are well-established prognostic factors for patients with HF: age; male sex; New York Heart Association functional class III/IV; history of hypertension, diabetes, and atrial fibrillation; systolic blood pressure at discharge; and serum albumin, hemoglobin, creatinine, sodium, potassium, and log-transformed BNP levels measured at the time of discharge. To clarify the association between prognosis and changes in FMHF class prescribed, we further included ACEi/ARB, BB, and MRA prescriptions at discharge as adjustment variables. Multiple imputations were used to account for missing covariate data. We created 20 datasets using a chained-equations procedure^23^. Parameter estimates were obtained for each dataset and subsequently combined to produce an integrated result using the method described by Barnard and Rubin^24^.

Statistical significance was considered at a *P*-value of < 0.05. All data were analyzed using R software version 3.6.3 (R Foundation for Statistical Computing, Vienna, Austria; ISBN 3-900051-07‐0, URL https://www.R-project.org).

## Results

Among all enrolled patients (n = 7,073), patients with missing data on FMHF at admission, discharge, or both (n = 453), LVEF ≥ 50% (n = 3,289), and no prior history of HF (n = 2,218) were excluded; finally, 1,113 patients were analyzed in the study (Supplementary Fig. S1). The prescription rates for ACEi/ARB, BB, and MRA were 55.5%, 67.3%, and 32.6% at admission and 67.6%, 84.2%, and 47.5% at discharge, respectively. Baseline characteristics are summarized in Table 1, and vital signs, medications, and laboratory data on admission are presented in Supplementary Table S2. There were no differences in age, male sex, LVEF, and BNP level between the three groups. Among all groups, the increased group had higher hemoglobin, lower creatinine and blood nitrogen urea levels, and higher prevalence of FMHF prescription at discharge.

**Table 1:** Baseline characteristics stratified by the modification pattern of FMHF during hospitalization.

Variables	Increased	Unchanged	Decreased	*P*-value
	N = 413	N = 607	N = 93	
Age (years)	76 [65–83]	76 [67–82]	76 [69–82]	0.862
Male sex, n (%)	277 (67.1)	397 (65.4)	66 (71.0)	0.543
**Clinical demographics**				
Systolic blood pressure at discharge (mmHg)	109 ± 17	108 ± 17	106 ± 17	0.146
Diastolic blood pressure at discharge (mmHg)	62 ± 11	61 ± 11	58 ± 10	0.013
Heart rate at discharge (beats/min)	72 ± 14	71 ± 12	74 ± 14	0.051
NYHA class ≥ III at discharge, n (%)	65 (16.1)	125 (20.7)	23 (25.0)	0.068
Never smoker, n (%)	236 (57.1)	362 (59.6)	53 (57.0)	0.696
**Echocardiography**				
Left ventricular ejection fraction (%)	32 ± 9	33 ± 9	33 ± 9	0.463
Left ventricle end-diastolic diameter (mm)	58 ± 9	59 ± 10	59 ± 9	0.233
Left ventricle end-systolic diameter (mm)	49 ± 10	50 ± 12	50 ± 10	0.640
Left atrial diameter (mm)	46 ± 9	47 ± 10	47 ± 9	0.042
**Comorbidities, n (%)**				
Hypertension	270 (65.4)	372 (61.3)	65 (69.9)	0.169
Diabetes	168 (40.7)	257 (42.3)	48 (51.6)	0.155
Dyslipidemia	179 (43.3)	274 (45.1)	46 (49.5)	0.549
Atrial fibrillation	198 (47.9)	286 (47.1)	37 (39.8)	0.354
Chronic obstructive pulmonary disease	33 (8.0)	39 (6.5)	6 (6.5)	0.646
Coronary artery disease	167 (40.4)	247 (40.7)	39 (41.9)	0.965
**Medications at discharge, n (%)**				
ACEi/ARBs	333 (80.6)	386 (63.6)	34 (36.6)	< 0.001
Beta-blockers	373 (90.3)	511 (84.2)	54 (58.1)	< 0.001
Aldosterone blockers	258 (62.5)	254 (41.8)	17 (18.3)	< 0.001
Loop diuretics	351 (85.0)	530 (87.5)	75 (80.6)	0.163
Calcium channel blockers	76 (20.7)	124 (22.6)	13 (14.4)	0.204
Statins	151 (36.6)	260 (43.0)	35 (37.6)	0.107
**Laboratory data at discharge**				
Hemoglobin (g/dL)	12.2 ± 2.2	11.8 ± 2.1	11.4 ± 2.1	< 0.001
Albumin (g/dL)	3.5 ± 0.5	3.6 ± 0.6	3.4 ± 0.6	0.058
BUN (mg/dL)	25.1 [18.6–37.0]	28.2 [20.3–41.2]	32.7 [19.4–45.7]	0.001
Creatinine (mg/dL)	1.2 [0.9–1.6]	1.3 [1.0–1.8]	1.5 [1.1–2.2]	< 0.001
eGFR (mL/min/1.73 m^2^)	56.6 [39.2–76.9]	48.8 [33.0–69.0]	43.6 [29.2–59.3]	< 0.001
eGFR < 60 mL/min/1.73 m^2^, n (%)	224 (54.6)	394 (65.0)	69 (75.0)	< 0.001
Sodium (mEq/L)	139 [136–141]	138 [136–140]	138 [135–140]	0.061
Potassium (mEq/L)	4.4 ± 0.5	4.4 ± 1.5	4.5 ± 0.6	0.658
BNP (pg/dL)	383.3 [199.1–648.8]	387.0 [192.9–609.2]	368.5 [220.0–832.8]	0.478
Length of hospital stay (days)	16 [11–26]	15 [10–24]	21 [10–37]	0.013

*ACEi* angiotensin-converting enzyme inhibitor, *ARB* angiotensin II receptor blocker, *BNP* brain natriuretic peptide, *BUN* blood urea nitrogen, *eGFR* estimated glomerular filtration rate, *FMHF* foundational medications for heart failure, *NYHA* New York Heart Association.

**Figure 1:**
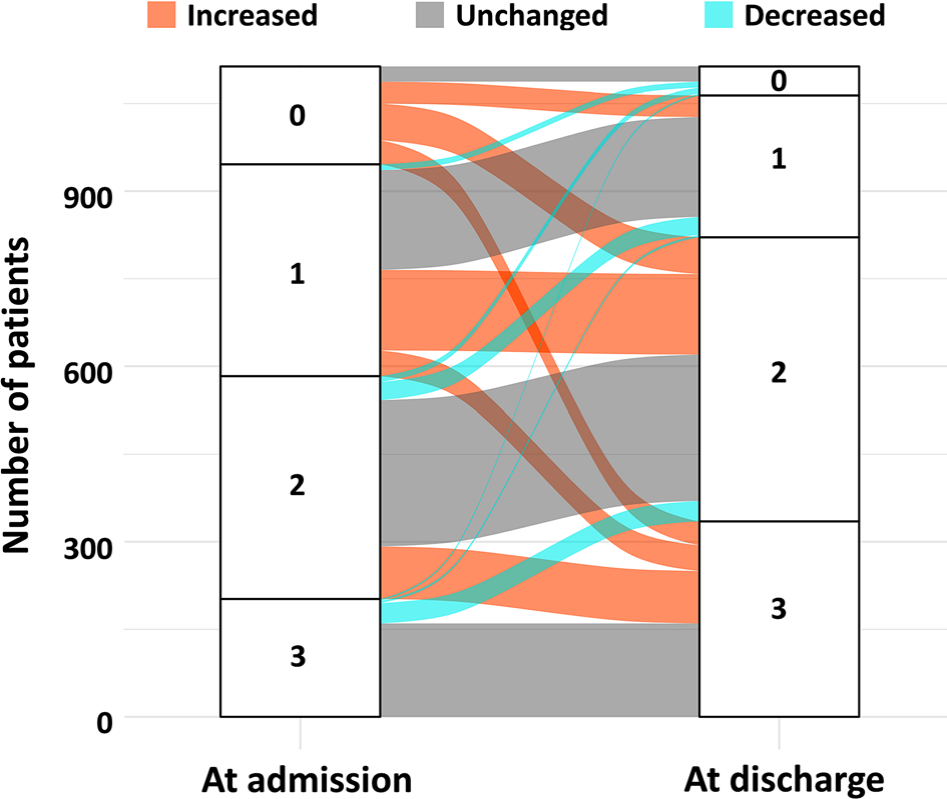
Change in the number of prescribed FMHF classes during hospitalization Alluvial plot showing the number of patients who remained in the same number of FMHF categories or transitioned to a different category from admission to discharge. *FMHF* foundational medications for heart failure.

At 1-year follow-up, the primary endpoint occurred in 482 patients. All-cause death was observed in 183 patients, out of whom 131 were cardiovascular-related deaths. Figure 1 shows the modification pattern of FMHF prescribed during hospitalization. Although most of the patients with no FMHF at admission had at least one FMHF and most of the patients prescribed three FMHF at admission maintained all three FMHF at discharge, only 50.1% and 23.6% of patients prescribed one FMHF and two FMHF, respectively, at admission had an increased number of FMHF prescriptions at discharge.

**Figure 2:**
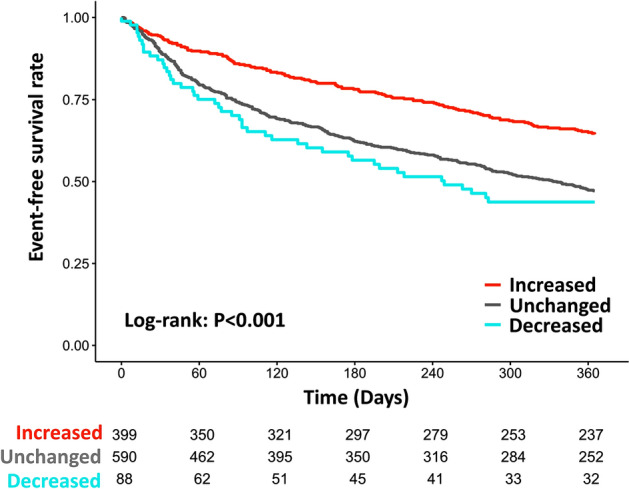
Kaplan–Meier curves stratified by changes in the number of prescribed FMHF from admission to discharge. There was a significant difference in survival rates between the three groups (log-rank *P* < 0.001).*FMHF* foundational medications for heart failure.

**Figure 3:**
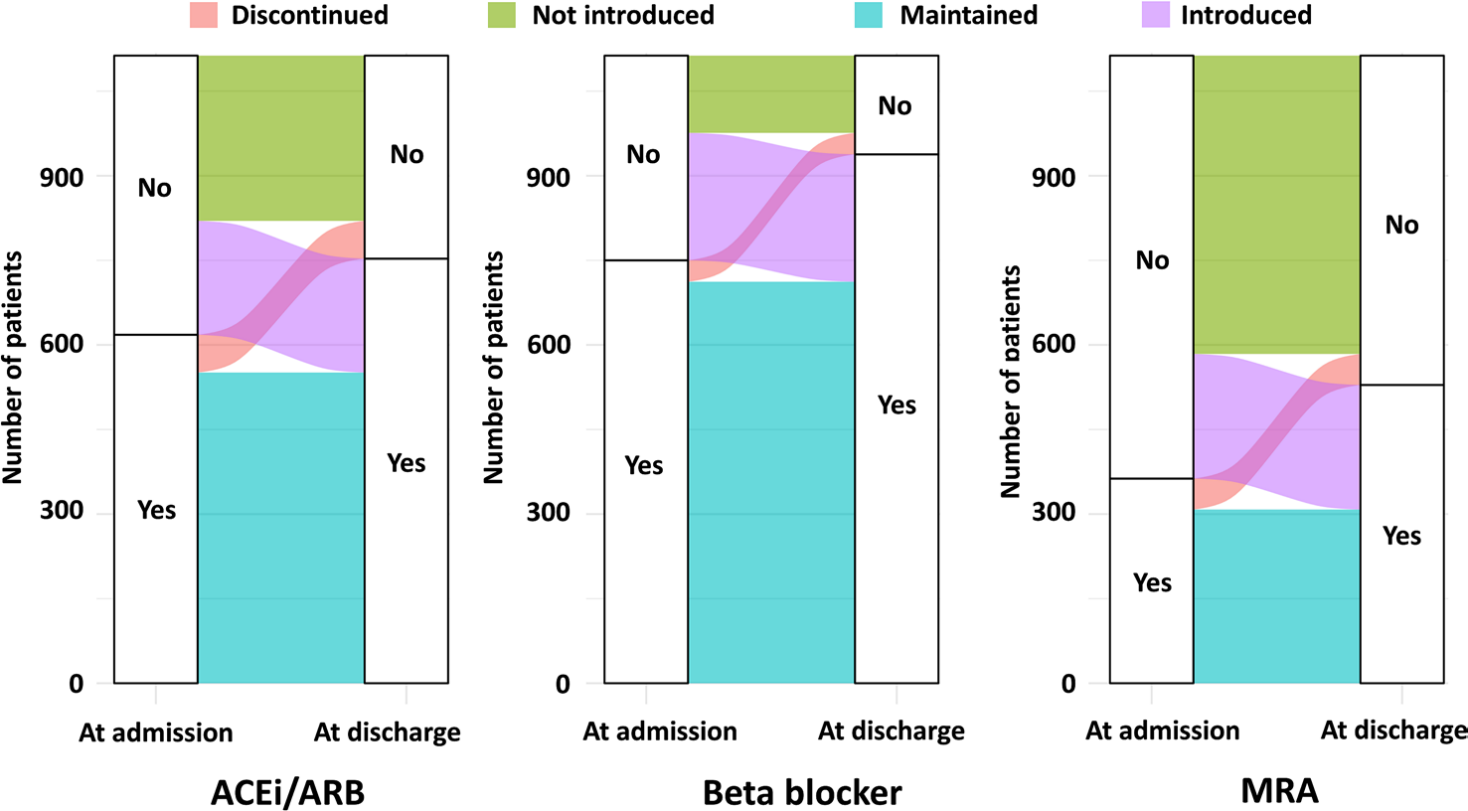
Modification pattern of each FMHF during hospitalization. Alluvial plot showing the changes in the prescription patterns of ACEi/ARB, beta-blocker, and MRA from admission to discharge. *ACEi* angiotensin-converting enzyme inhibitor, *ARB* angiotensin II receptor blocker, *FMHF* foundational medications for heart failure, *MRA* mineralocorticoid receptor antagonist.

Kaplan–Meier curves revealed that the increased group was significantly associated with better outcomes (log-rank *P* < 0.001) (Fig. 2). Furthermore, the study patients were stratified according to the number of FMHF classes prescribed at admission. The increased group was associated with better outcomes in all categories, except those already on three FMHF classes at admission (Supplementary Fig. S2). The modification pattern of each FMHF during hospitalization was divided into four groups: discontinued, maintained, not introduced, and introduced (Fig. 3). Patients who were not on FMHF at the time of admission were categorized as introduced or not introduced according to whether the patient was on or off FMHF at discharge. If the patient was on FMHF at admission, the patient was categorized as discontinued or maintained according to whether the patient was on FMHF at discharge. Although most patients on FMHF at admission were discharged without discontinuation, only 40.8%, 62.3%, and 29.4% of patients not on ACEi/ARB, BB, and MRA, respectively, at the time of admission were prescribed FMHF at discharge. Furthermore, we evaluated the association between the modification pattern and prognosis for each FMHF medication and found that the introduction of FMHF was significantly associated with better outcomes in all classes (Supplementary Fig. S3).

To evaluate whether the association between changes in the number of FMHF classes and prognosis was independent of other covariates, unadjusted and adjusted Cox proportional hazard analyses were performed. Univariate Cox proportional hazard analysis showed that the increased group was associated with a lower incidence of the primary outcome than the unchanged and decreased groups, and this association was consistent even after adjustment for possible prognostic factors, including ACEi/ARB, BB, and MRA prescriptions at discharge (increased vs. unchanged group: hazard ratio [HR], 0.57; 95% confidence interval [CI], 0.46–0.71; *P* < 0.001; increased vs. decreased group: HR, 0.59; 95% CI, 0.41–0.85; *P* = 0.005) (Table 2). In the sensitivity analysis, we added the prescription of loop diuretics on admission and an addition of loop diuretics during hospitalization in the multivariate analysis because these factors might reflect the severity of HF. After including these two factors, the result was not changed (increased vs. unchanged group: HR, 0.62; 95% CI, 0.50–0.78; *P* < 0.001; increased vs. decreased group: HR, 0.64; 95% CI, 0.44–0.93; *P* = 0.019).

**Table 2:** Unadjusted and adjusted Cox regression for combined endpoint.

Group	Unchanged group as reference						Decreased group as reference					
	Unadjusted Cox regression			Adjusted Cox regression*			Unadjusted Cox regression			Adjusted Cox regression*		
	HR	95% CI	*P*-value	HR	95% CI	*P*-value	HR	95% CI	*P*-value	HR	95% CI	*P*-value
Unchanged	Ref			Ref			0.86	0.63–1.17	0.333	1.03	0.75–1.43	0.843
Increased	0.58	0.47–0.70	< 0.001	0.57	0.46–0.71	< 0.001	0.49	0.35–0.69	< 0.001	0.59	0.41–0.85	0.005
Decreased	1.16	0.85–1.59	0.338	0.97	0.70–1.34	0.843	Ref			Ref		

*Adjusted for age, male sex, New York Heart Association functional class III/IV, history of hypertension, diabetes, atrial fibrillation, systolic blood pressure at discharge, serum albumin, hemoglobin, creatinine, sodium, potassium, and log-transformed BNP measured at the time of discharge; prescription of ACEi/ARB, BB, and MRA at discharge.

## Discussion

In this study, we investigated the modification patterns of FMHF and their impact on outcomes in patients with HFr/mrEF who were hospitalized because of exacerbation of HF. We showed that increasing the number of FMHF classes during hospitalization was associated with lower incidence of 1-year adverse events.

In line with the results of a prior study^25^, we found that the prescription rates of all FMHF classes increased even in patients already diagnosed with HF before admission. Overall, the prescription rates of ACEi/ARB, BB, and MRA increased by 12.1% (from 55.5 at admission to 67.6% at discharge), 16.9% (from 67.3 to 84.2%), and 14.9% (from 32.6 to 47.5%) during hospitalization, respectively. These results are not different from those of a previous study. In the OPTIMIZE-HF registry^26^, which is a large-scale hospital-based registry in the US, the prescription rates of ACEi/ARB, BB, and MRA in patients with LVEF < 50% increased by 16.2% (54.3–70.4%), 15.7% (55.4–71.1%), and 6.9% (8.9–15.8%) during hospitalization, respectively. The increase in the MRA prescription rate from admission to discharge was higher in our study sample than in the OPTIMIZE-HF group. This difference could be explained by the study population. The OPTIMIZE-HF group included patients hospitalized with both new-onset and worsening pre-existing HF, while our study included only patients with a history of HF and thus could be more saturated with an advanced stage of HF. Furthermore, this might be because the OPTIMIZE-HF registry was performed before the additive prognostic importance of MRA on ACEi/ARB and BB was shown in the EMPHASIS-HF trial publication in 2011^27^. Nevertheless, our results show that the rate of introduction of MRA is the lowest among the three FMHF, which is in line with other reports demonstrating a low prescription rate of MRA in HF with reduced ejection fraction^5,28,29^.

However, some patients discontinued FMHF, although they were few. The discontinuation rates were similar in the sub-study of the CHAMP-HF registry, which evaluated the prescription rates of medications for patients with a history of HF at 12 months after enrollment^25^. In our study, 10.8%, 5.0%, and 15.1% of those on ACEi/ARB, BB, and MRA at admission discontinued each medication, respectively, while in the CHAMP-HF registry, they were 18.0%, 5.0%, and 21.1%, respectively. Although the actual reasons for the discontinuation of FMHF are unclear, the creatinine level was higher in the decreased group than in the increased FMHF group. There were no significant differences in age, sex, comorbidities, systolic blood pressure, or heart rate at discharge among the three groups. Patients hospitalized with acute decompensated HF might have unstable hemodynamics, worsening renal function, or electrolyte changes, and thus discontinuation of FMHF might be unavoidable.

Prior studies have demonstrated that the initiation or continuation of FMHF during hospitalization was both safe and efficacious. The OPTIMIZE-HF registry showed that BB initiation during hospitalization was associated with lower mortality or rehospitalization rates than no initiation in patients with left ventricular systolic dysfunction^30^. Moreover, in-hospital BB withdrawal was independently associated with a higher mortality risk than BB continuation^31^. A meta-analysis including three observational studies and one randomized clinical trial demonstrated an increased risk of combined short-term rehospitalization or death when BB was withdrawn in patients hospitalized for acute decompensated HF^32^. Furthermore, the GWTG-HF registry showed that in-hospital withdrawal of ACEi/ARB for patients with HFrEF was independently associated with a higher risk of 1-year mortality than continued withdrawal^33^. In the CHAMP-HF registry, ACEi/ARB, angiotensin receptor neprilysin inhibitors (ARNI), BB, and MRA de-escalation or discontinuation after HF hospitalization were associated with an increased risk of all-cause mortality^25^. Contrastingly, to the best of our knowledge, no study has evaluated the association between changes in the number of prescribed FMHF classes during hospitalization and prognosis in patients with HF. In this study, we found the possibility that an increase in the number of FMHF classes was significantly associated with better post-discharge prognosis in hospitalized patients who did not have de novo HF. It should be highlighted that this association was independent of FMHF prescription at discharge, implying that changes in the number of FMHF classes might be associated with prognosis. This finding could show the importance of increasing the number of FMHF classes during hospitalization due to worsening HF, and patients who were discharged without increasing the number of FMHF were associated with poor prognosis. Although we did not consider the impact of the dose of each FMHF, our study results are also in line with those of a previous study that demonstrated that even a low starting dose of each class of FMHF was associated with decreased mortality^9^.

Real-world registries revealed that the prescription rates of FMHF were not high even in patients with HFrEF (CHAMP-HF: prescription rates for ACEi/ARB/ARNI, BB, and MRA were 73.4%, 67.0%, and 33.4%; VICTORIA registry: prescription rates for ACEi/ARB/ARNI, BB, and MRA were 65.0%, 75.1%, and 40.5%)^5,34^. Thus, there is a scope for improvement in the FMHF prescription rates; furthermore, we assume that the current analysis can highlight the importance of FMHF and assist in increasing the prescription rates from a different perspective. Moreover, this study primarily focused on patients with HF who required hospitalization due to worsening, which makes our study unique. Therefore, with the use of real-world data, this study can suggest the benefit of improved prescription rates in improving the outcomes. However, clinicians should carefully evaluate the tolerability of FMHF and avoid reckless prescription of FMHF. In patients who already used all three FMHF (ACEi/ARB, BB, and MRA) at admission, we could not explore the impact of adding or switching to recently developed HF medications, including ARNI and sodium-glucose co-transporter-2 inhibitors, as our study cohort data were registered before these drugs became available in Japan, and further interventions may be required for the population. However, given that patients tested in the studies showing the prognostic benefit of these relatively novel drugs have already been well treated with ACEi/ARB, BB, and MRA^35–37^, it is likely that increasing the number of FMHF patients using these drugs can improve the prognosis of hospitalized patients who are already on ACEi/ARB, BB, and MRA^38,39^. However, this hypothesis needs to be evaluated in future studies.

This study has several limitations. First, as this was a post-hoc analysis of observational studies, some prognostically relevant variables such as exercise capacity could not be obtained. Moreover, in the current study, the decreased group had lower blood pressure, heart rate, and hemoglobin level; higher potassium level; worse renal function; and longer length of hospital stay than the other two groups. Since it could be suggested that the decreased group has severer HF and poorer tolerability of FMHF and is associated with worse post-discharge outcomes, we could not infer a causal relationship between changes in FMHF and prognosis. Thus, clinicians should carefully assess the tolerability of FMHF and should not erratically increase the number of FMHF. Second, we investigated the modification patterns of FMHF during hospitalization; however, we did not consider whether FMHF changed after discharge. We had no data on vital signs, heart rhythm, electrolytes, or renal function during the follow-up period, and changes in FMHF were made at the discretion of clinicians, although these factors could affect changes in FMHF after discharge. However, a recent multicenter prospective registry focusing on FMHF for HFrEF showed that the prescription rate and dose did not change significantly over time during a 1-year follow-up^29^. Nevertheless, this is an important limitation of our observational study, and our study results should be carefully interpreted. Third, we did not have data on dose modification for each FMHF and did not consider its impact on the results. Thus, the question of whether we should attempt to maximize the total number of FMHF rather than dose titrations should be addressed in future studies. Fourth, we analyzed both HFrEF and HFmrEF, although the prognostic impact of FMHF has not been tested in randomized clinical trials for patients with HFmrEF. However, a series of studies have already shown that FMHF is likely associated with improved prognosis in patients with HFmrEF^40–44^, and the latest European Society of Cardiology and the American College of Cardiology, American Heart Association, and Heart Failure Society of America guidelines provide IIb recommendations to FMHF for patients with HFmrEF in terms of reducing HF hospitalization and death^2,3^. Finally, we included patients who discontinued some medications and initiated other medications in each group, which might complicate the interpretation of the results. However, this study was limited to a small number of cases.

In conclusion, for patients hospitalized with HFr/mrEF, an increase in the number of FMHF classes during HF hospitalization may be associated with lower incidence of 1-year adverse events. Our observational study may have implications for the FMHF optimization approach for patients with HF. Further studies are warranted to investigate the association between the number of FMHF classes and prognosis.

## Supplementary Information

Supplementary Information.

## Data Availability

The datasets generated during and/or analyzed during the current study are not publicly available due to their containing information that could compromise the privacy of research participants but are available from the corresponding author on reasonable request.
