# Supplementary material for: Association between class of foundational medication for heart failure and prognosis in heart failure with reduced/mildly reduced ejection fraction
Source: Sci Rep. 2022 Oct 5;12:16611. doi: 10.1038/s41598-022-20892-3 (PMC9534994; doi:10.1038/s41598-022-20892-3)
Supplement: Supplementary file 1 — Supplementary Information. [file 41598_2022_20892_MOESM1_ESM.pdf]

Supplementary Information

Association between class of foundational medication for heart failure and prognosis in heart failure with reduced/mildly reduced ejection fraction

Miyuki Ito, Daichi Maeda, Yuya Matsue, Yasuyuki Shiraishi, Taishi Dotare, Tsutomu Sunayama, Kazutaka Nogi, Makoto Takei, Tomoya Ueda, Maki Nogi, Satomi Ishihara, Yasuki Nakada, Rika Kawakami, Nobuyuki Kagiyama, Takeshi Kitai, Shogo Oishi, Eiichi Akiyama, Satoshi Suzuki, Masayoshi Yamamoto, Keisuke Kida, Takahiro Okumura, Yuji Nagatomo, Takashi Kohno, Shintaro Nakano, Shun Kohsaka, Tsutomu Yoshikawa, Yoshihiko Saito, Tohru Minamino

Supplementary Fig. S1. Study flow chart

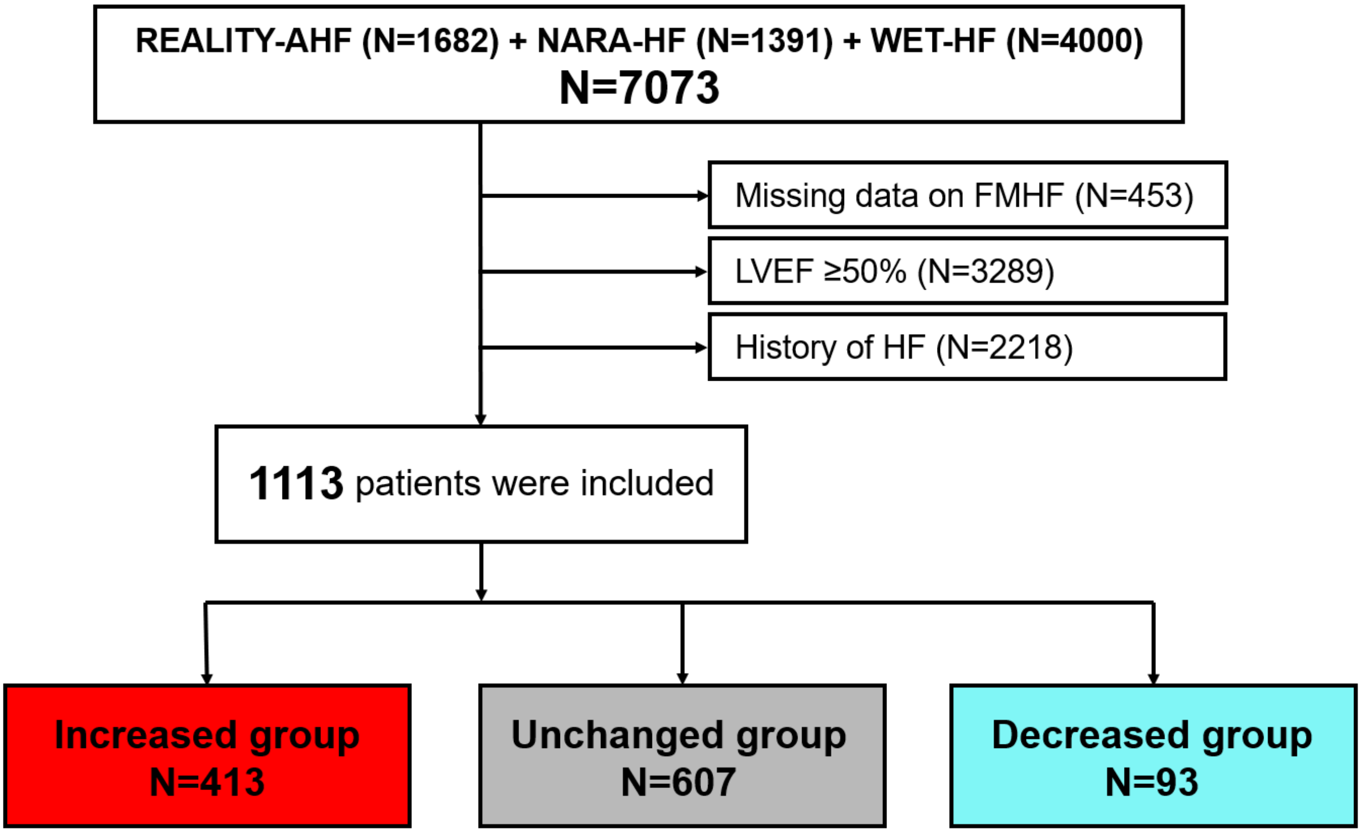

Supplementary Fig. S2. Kaplan–Meier curves stratified by changes in the number of prescribed foundational medications for heart failure (FMHF) from admission to discharge for patients on (A) no, (B) 1, (C) 2, and (D) 3 FMHF at admission

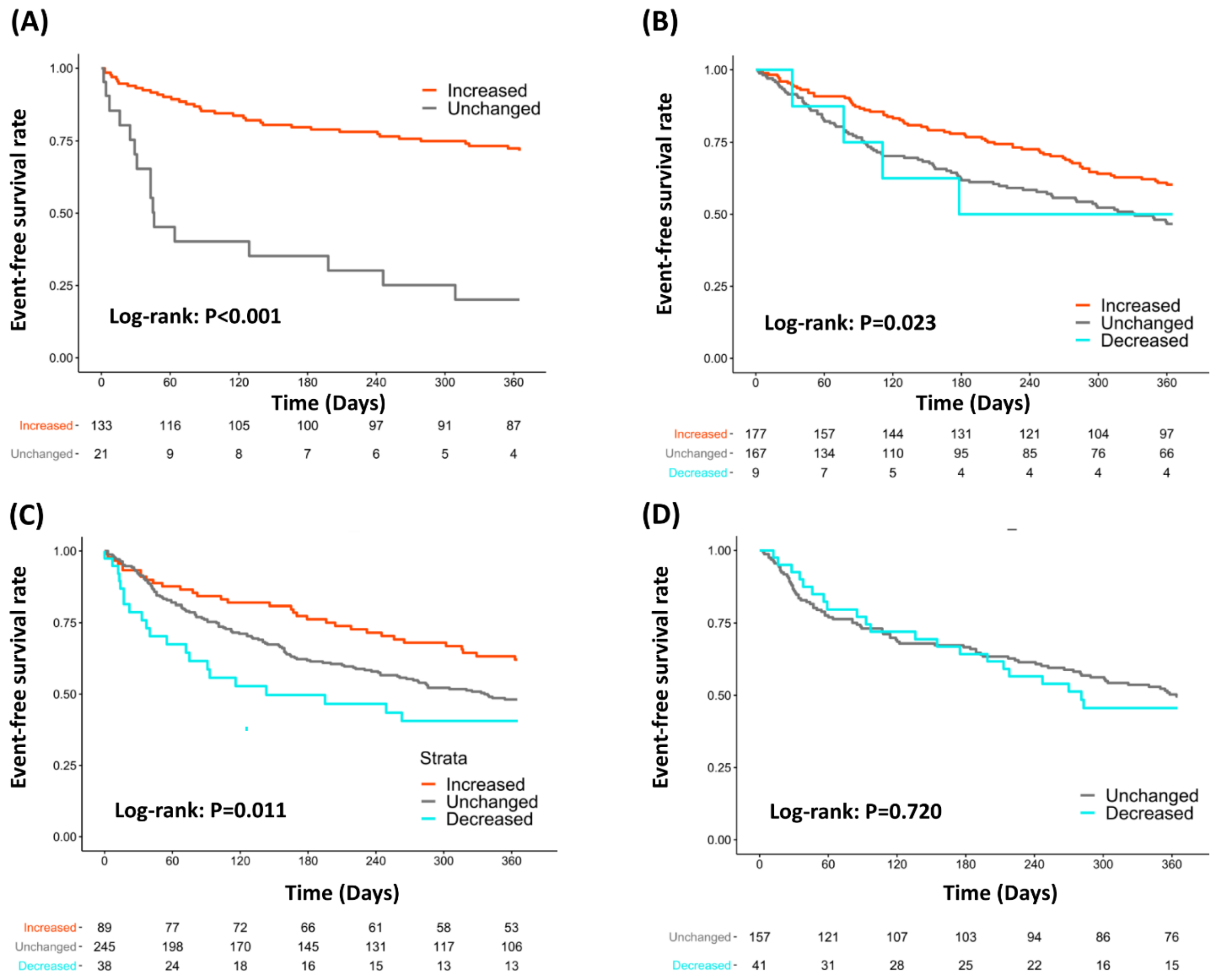

Supplementary Fig. S3. Kaplan–Meier curves showing the association between the modification pattern and prognosis for each medication of FMHF

(A) ACEi/ARB

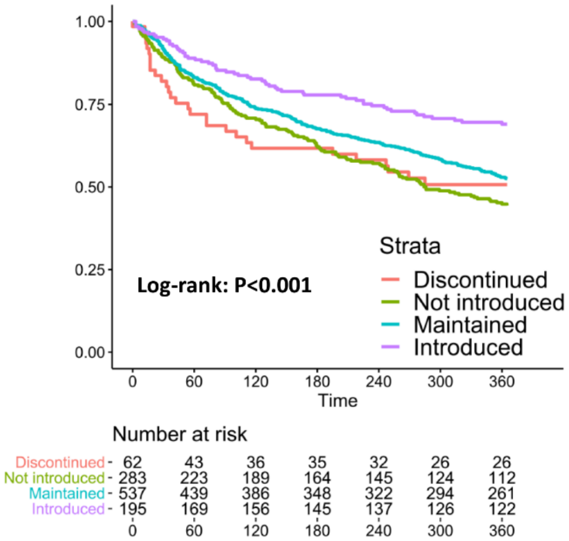

(B) Beta blocker

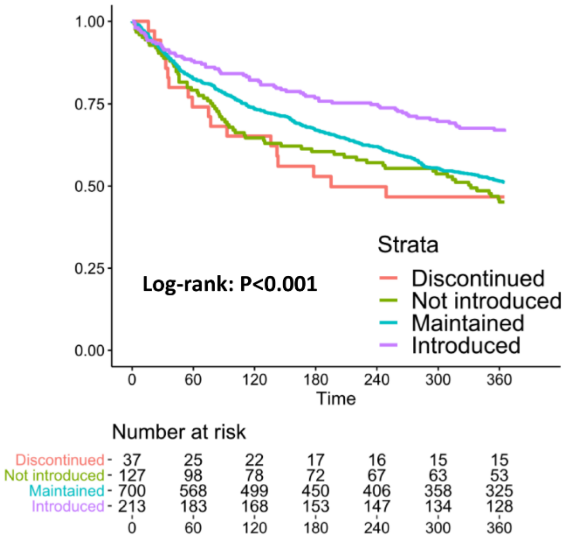

(C) MRA

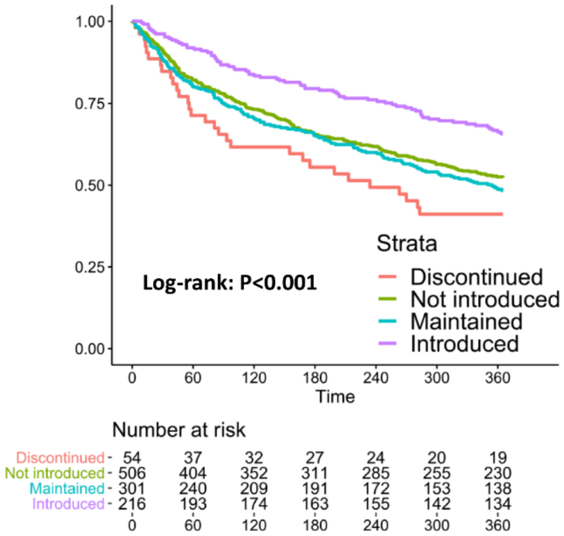

Supplementary Table S1. Information on REALITY-AHF, NARA-HF, and WET-HF

Registries	Information
REALITY-AHF	REALITY-AHF (Registry Focused on Very Early Presentation and Treatment in Emergency Department of Acute Heart Failure) is a prospective multicenter study that investigated the relationship between time to treatment and prognosis in patients with HF in the very acute phase.[1] Between August 2014 and December 2015, 1,682 consecutive patients hospitalized for acute HF through the emergency department of the participating hospitals were registered. Patients with an acute coronary syndrome, previous heart transplantation, chronic peritoneal dialysis, hemodialysis, or acute myocarditis were excluded. Clinical data, including the types and doses of intravenous medications, were obtained until 48 h after arrival at the emergency department. Oral medications taken within 48 h and at discharge were also obtained. At the emergency department, baseline physical findings and blood samples were evaluated for all patients. Laboratory data and echocardiographic findings were obtained from the emergency department and steady-state phases before discharge.
NARA-HF	The Nara Registry and Analyses for Heart Failure (NARA-HF) cohort retrospectively registered consecutive 1,391 patients with acute HF hospitalized at Nara Medical University Hospital from January 2007 to March 2011 and prospectively registered patients from April 2011 to December 2018. [2,3] Patients with acute coronary syndrome, myocarditis, or acute pulmonary embolism were excluded. Blood tests and echocardiography were performed on admission and in a clinically compensated state before discharge. Information regarding oral medications was also collected upon admission and discharge.
WET-HF Registry	The West Tokyo Heart Failure (WET-HF) registry is an ongoing, prospective, multicenter cohort study that evaluates the clinical backgrounds and prognosis of patients hospitalized with acute HF. [4] Between January 2008 and August 2017, 4,000 consecutive patients hospitalized with acute-onset HF or a change in the clinical signs of HF that required urgent therapy were registered. Patients with acute coronary syndromes were excluded from this study. Clinical data, including demographic information, medical history, laboratory tests at several time points during hospitalization, medication, and clinical outcomes during hospitalization and after discharge, were obtained.

References:

[1] Matsue, Y. *et al.* Time-to-furosemide treatment and mortality in patients hospitalized with acute heart failure. *J. Am. Coll. Cardiol.* **69**, 3042-3051 (2017).
[2] Ishihara, S. *et al.* Incidence and clinical significance of 30-day and 90-day rehospitalization for heart failure among patients with acute decompensated heart failure in Japan- From the NARA-HF study. *Circ. J.* **84**, 194-202 (2020).
[3] Misumi, K. *et al.* Usefulness of incorporating hypochloremia into the get with the guidelines-heart failure risk model in patients With acute heart failure. *Am. J. Cardiol.* **162**, 122-128 (2022).
[4] Shiraishi, Y. *et al.* Validation of the Get With the Guideline-Heart Failure risk score in Japanese patients and the potential improvement of its discrimination ability by the inclusion of B-type natriuretic peptide level. *Am. Heart J.* **171**, 33-39 (2016).

Supplementary Table S2. Baseline characteristics on admission

Variables	Increased N=413	Unchanged N=607	Decreased N=93	P-value
Vital signs on admission				
Systolic blood pressure (mmHg)	137±32	130±32	128±36	<0.001
Diastolic blood pressure (mmHg)	82±23	77±21	74±22	<0.001
Heart rate (beats/min)	100±29	89±24	87±26	<0.001
Medications at admission, n (%)				
ACEi/ARB	143 (34.6)	397 (65.4)	78 (83.9)	<0.001
Beta-blocker	161 (39.0)	505 (83.2)	84 (90.3)	<0.001
Aldosterone blocker	58 (14.0)	249 (41.0)	56 (60.2)	<0.001
Loop diuretic	237 (57.5)	499 (82.3)	81 (87.1)	<0.001
Calcium channel blocker	84 (22.5)	119 (20.7)	22 (24.4)	0.641
Statin	110 (29.5)	247 (43.0)	37 (41.1)	<0.001
Laboratory data at admission				
Hemoglobin (g/dL)	12.4±2.4	11.8±2.2	11.4±2.1	<0.001
Albumin (g/dL)	3.6±0.5	3.6±0.6	3.5±0.6	0.284
BUN (mg/dL)	24.0 [18.0–34.0]	28.0 [20.0–40.8]	31.5 [21.0–49.0]	<0.001
Creatinine (mg/dL)	1.2 [0.9–1.6]	1.3 [1.0–1.9]	1.4 [1.1–2.3]	<0.001
eGFR (mL/min/1.73 m^2)	57.8 [40.0–77.8]	48.6 [31.6–68.9]	42.0 [27.1–61.9]	<0.001
eGFR <60 mL/min/1.73 m^2, n (%)	221 (53.5)	406 (66.9)	68 (73.1)	<0.001
Sodium (mEq/L)	139 [137–142]	139 [137–141]	138 [136–141]	0.271
Potassium (mEq/L)	4.3±0.7	4.4±0.7	4.5±0.7	0.010
BNP (pg/dL)	970.5 [575.7–1719.5]	859.7 [490.0–1586.1]	1017.5 [671.2–1882.6]	0.082

ACEi, angiotensin-converting enzyme inhibitor; ARB, angiotensin II receptor blocker; BNP, brain natriuretic peptide; BUN, blood urea nitrogen; eGFR, estimated glomerular filtration rate
